# An epidermal sEMG tattoo-like patch as a new human–machine interface for patients with loss of voice

**DOI:** 10.1038/s41378-019-0127-5

**Published:** 2020-03-09

**Authors:** Huicong Liu, Wei Dong, Yunfei Li, Fanqi Li, Jiangjun Geng, Minglu Zhu, Tao Chen, Hongmiao Zhang, Lining Sun, Chengkuo Lee

**Affiliations:** 1 School of Mechanical and Electric Engineering, Jiangsu Provincial Key Laboratory of Advanced Robotics, Soochow University, 215123 Suzhou, China; 2 Department of Electrical & Computer Engineering, National University of Singapore, 4 Engineering Drive 3, Singapore, 117576 Singapore; 3 National University of Singapore Suzhou Research Institute (NUSRI), Suzhou Industrial Park, 215123 Suzhou, China

**Keywords:** Nanoscale materials, Nanoscale devices, Nanoscale materials, Nanoscale devices, Nanoscale materials

## Abstract

Throat cancer treatment involves surgical removal of the tumor, leaving patients with facial disfigurement as well as temporary or permanent loss of voice. Surface electromyography (sEMG) generated from the jaw contains lots of voice information. However, it is difficult to record because of not only the weakness of the signals but also the steep skin curvature. This paper demonstrates the design of an imperceptible, flexible epidermal sEMG tattoo-like patch with the thickness of less than 10 μm and peeling strength of larger than 1 N cm^−1^ that exhibits large adhesiveness to complex biological surfaces and is thus capable of sEMG recording for silent speech recognition. When a tester speaks silently, the patch shows excellent performance in recording the sEMG signals from three muscle channels and recognizing those frequently used instructions with high accuracy by using the wavelet decomposition and pattern recognization. The average accuracy of action instructions can reach up to 89.04%, and the average accuracy of emotion instructions is as high as 92.33%. To demonstrate the functionality of tattoo-like patches as a new human–machine interface (HMI) for patients with loss of voice, the intelligent silent speech recognition, voice synthesis, and virtual interaction have been implemented, which are of great importance in helping these patients communicate with people and make life more enjoyable.

## Recovering lost voices

Tattoo-like wearable electronic patches could help patients who have lost the capacity for speech to communicate once again. The aftermath of surgeries for mouth and throat cancers can result in permanent voice loss, but the information needed to decode speech can still be recovered from the movements of facial muscles. Researchers led by Hongmiao Zhang and Lining Sun at China’s Soochow University and Chengkuo Lee at the National University of Singapore have developed flexible sensors that can be applied to the skin to capture this information. In a series of tests with human volunteers, they demonstrated that vocalization-associated muscle activity collected from these patches could be computationally decoded with up to 92% accuracy. Similar devices could eventually be coupled to voice synthesizers or other devices to help restore normal speech after cancer surgery.

## Graphical abstract

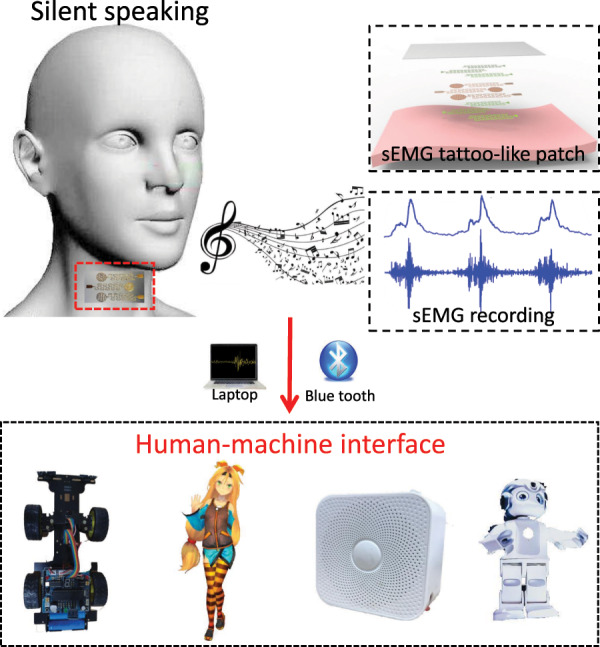

## Introduction

Speech is an important means by which humans communicate and express their emotions and opinions. In the United States, 53,000 adults are expected to be diagnosed with oral and oropharyngeal cancer in 2019^1^. Treatment can involve surgical removal of the tumor, radiation therapy, and chemotherapy, leaving patients with facial disfigurement as well as temporary or permanent loss of voice, which greatly hinders daily patient communication^2^.

Surface electromyography (sEMG) is a bioelectrical signal emitted from neuromuscular activity and can be recorded by electrodes on the surface of the human skeletal muscle, which are widely used in clinical medicine, kinematics, etc.^3–5^. Studies have confirmed that sEMG generated by the muscles of the face and the lower jaw contains useful voice information related to speaking^6,7^. Different muscle motion patterns are produced by the contraction of different muscle groups, and the accompanying sEMG signals are different. In silent speech, the muscle groups of the face and the lower jaw correspond to different motion patterns, so it is entirely possible to distinguish the motion patterns from different sEMG signals and identify the internal speech information. Since the 1980s, sEMG from some smaller muscles associated with speech has been analyzed, and the results are promising. Michael S. Morse et al. used four-channel facial sEMG to recognize 10 English words. Their best accuracy rate was 58.0%, using the magnitude as the sEMG feature^8^. C. Jorgensen et al. used sEMG to perform six-word speech recognition in a real-time simulated environment, in which six words can be used as a control set for NASA's spacecraft Mars Cruiser. M. Janke et al. analyzed and compared sEMG generated from audible, whispered, and silent speech, indicating that speech recognition based on sEMG can be applied to silently mouthed speech^9^.

In fact, it is very difficult to acquire sEMG signals from the jaw and face by traditional sEMG electrodes because of not only the weakness of the signals but also the sharp skin curvature of this area. Traditional sEMG measurements use rigid electrodes coupled to the skin via electrolyte gels affixed with adhesive tape or straps. During the recording, the electrode sheet may be detached from the superficial skin, resulting from the drying of the gel and the movement of the muscle, leading to a decrease in measurement accuracy^10^. These defects also practically limit mounting locations to relatively flat regions of the body, such as the forehead, back, chest, forearm, or thigh. The ability to extend the traditional sEMG electrode to measure the sEMG of the jaw is deficient.

Owing to the recent advances and the rapid development in soft materials and micro-electromechanical system (MEMS) fabrication, the emerging field of wearable flexible electronic devices offers a technological solution to measure the sEMG of the face, jaw, neck, etc.^11–23^. One of the major trends is to apply textiles made of functional yearns and coatings or to use flexible materials to fabricate devices for detecting physiological signals^24–26^, conducting drug delivery^27^, and realizing intuitive human– machine interfaces^28–31^. Another trend is the thin-film technique for stretchable electronics and wearables, including epidermal sensors, the epidermal electronic system (EES), and electronic tattoos (e-tattoos), which have demonstrated a wide range of functionalities, including physiological sensing^32–43^, on-skin display^44^, ultraviolet (UV) detection^45^, transdermal therapeutics^34^, human–machine interface (HMI)^46^, prosthetic electronic skin^47^, and skin-adhesive rechargeable batteries^48,49^.

J. A. Rogers et al. proposed EES interfaces that can record sEMG signals from four different bimanual gestures of forearms to control a drone quadrotor^10^. Lu and colleagues proposed graphene electronic tattoo (GET) electrooculography (EOG) sensors to control a quadcopter wirelessly in real time to demonstrate the functionality of GET EOG sensors for the human–robot interface^50^. T. Ren et al. manufactured an intelligent laser-induced graphene artificial throat, which can not only generate sound but also detect sound in a single device^51^. H. Zhang et al. proposed a flexible hybrid device that can be attached to different parts of the body for the real-time monitoring of human physiological signals, such as respiratory information and the radial artery pulse^52^. It is apparent that using ultrathin dry electrodes instead of wet conductive gel silver/silver chloride (Ag/AgCl) electrodes to couple to human skin is beneficial for expanding the monitoring area for silent speech recognition and improving human comfort.

**Fig. 1:**
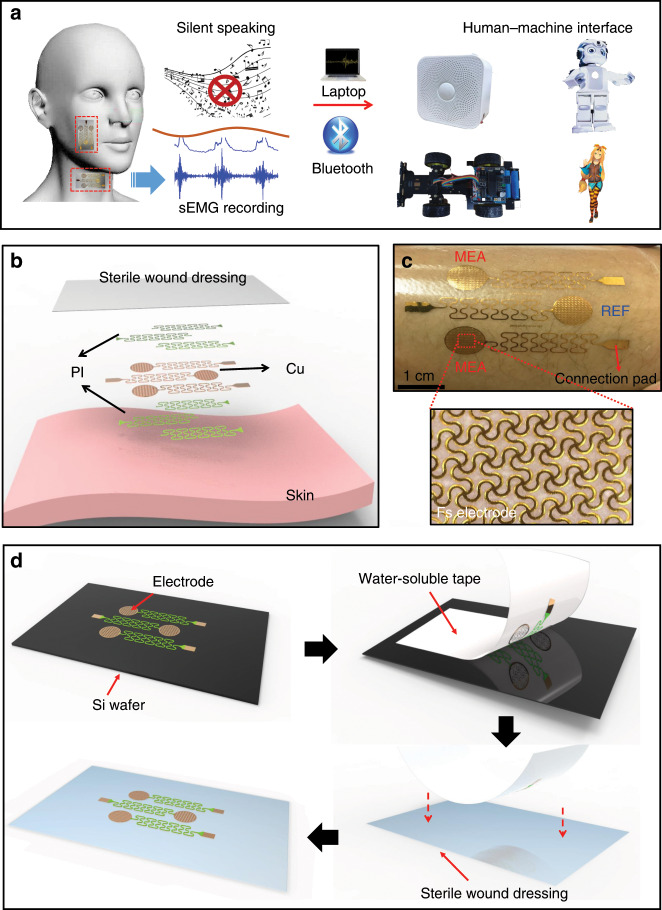
Introduction of the epidermal sEMG tattoo-like patch for patients with loss of voice. **a** Schematic drawing showing the control flow of the human–machine interface based on the epidermal sEMG tattoo-like patch for patient voice loss. **b** Layer-by-layer configuration of the epidermal sEMG electrode patch. **c** Epidermal sEMG tattoo-like patch mounted on the forearm. The configuration and the internal structure of the epidermal sEMG electrode, including two sensing electrodes (MEA) and a reference electrode (REF) of filamentary serpentine (FS) meshes, are shown. **d** Double-transfer process of the epidermal sEMG electrode from the wafer to the sterile wound dressing.

Generally, the ability to accurately and imperceptibly monitor health sEMG is vital for healthcare professionals and patients. The development of machine-learning techniques in data analysis offers much assistance in decoding sEMG signals, which dramatically enhance the reliability of information recognition and expand the application fields. This study illustrates an epidermal sEMG tattoo-like patch that can be laminated on the jaw and face without any additional adhesives and imperceptibly measures the sEMG from a patient who has lost his/her voice. Experiments are conducted, and the sEMG signals are collected when the tester speaks silently. Through wavelet decomposition and machine-learning algorithms, the frequently used action and emotion instructions can be recognized with high accuracy. To demonstrate the functionality of the epidermal sEMG tattoo-like patch for the human–machine interface, action instructions of silent speech recognition are used to control an intelligent car, and emotion instructions are used to control a Bluetooth speaker and virtual interaction in real time (Fig. 1a).

## Design of the epidermal sEMG tattoo-like patch

The electrode layout of the sEMG patch contributes to the magnitude of the electrical noise and crosstalk contamination, such as the electrode size, the interelectrode distance, and the electrode shape. As illustrated in Fig. 1b, the electrodes and interconnects are made of copper (Cu). The double-wave interconnected Cu wires incorporate polyimide (PI) coatings above and below each layer to physically encapsulate the Cu traces and minimize the bending-induced strains. The Cu traces are terminated at the exposed contact pads for the purpose of external connection and signal acquisition. As the European concerted sEMG group determined, the two measuring electrodes should provide separations of 20 mm, measured along the direction of the muscle fibers to obtain the optimal signals^10^. The filamentary serpentine (FS) mesh electrodes involve bare metal in direct contact with the skin. Figure 1c shows the epidermal sEMG tattoo-like patch mounted on the forearm. The patch consists of two measuring electrodes (MEA) and a reference electrode (REF) (400 nm in thickness and 9 mm in diameter) with FS meshes of 0.1 mm in width and 0.2 mm in radius of curvature. The interconnects are designed in a double-wave wire structure to enhance the local stretchability and deformability of the patch. The electrodes and interconnects are transferred onto a sterile wound dressing (polyurethane, Qingdao Hainuo, CHINA). The sterile wound dressing can provide not only excellent flexibility but also great adhesiveness to the muscles of the lower jaw and face without falling off. The layout and configuration yield soft and elastic responses to applied strains in a manner that both provides conformal contact to the skin and the ability to accommodate natural motions without mechanical constraints or interface delamination.

The fabrication process follows the micromachining procedures described in the “Experimental section” (see supplementary information, Fig. S1), in which all electrode-patterning processes are carried out on a silicon wafer. As illustrated in Fig. 1d, the double-transfer process by using a water-soluble tape (3 M, USA) releases the resulting device from the wafer to the sterile wound dressing. Specifically, the first step is to use the soluble tape to emancipate the epidermal sEMG electrodes from the silicon wafer. Then, the application of water dissolves the water-soluble tape after transferring the electrode to the sterile wound dressing.

## Results and discussion

### Mechanical characterization

The face and lower jaw of the human body typically have a fairly high skin curvature. However, the majority of the current physiological monitoring systems, including traditional sEMG electrodes, are made of rigid metal or hard materials and coupled to the skin via electrolyte gels. These rigid electrodes are difficult to apply to areas with a complex skin curvature and need to be affixed with adhesive tape or straps to prevent them from falling off. To overcome the drawbacks of traditional rigid electrodes and increase the comfort of the patients wearing them, imperceptible and flexible epidermal electrodes with good adhesiveness that can match the sharp curvatures of the jaw and face more closely are required. To test and verify the flexibility of the epidermal sEMG tattoo-like patch, a mechanical stage is used to stretch the patch, and the finite element method (FEM) is used to simulate the deformation and stress distribution of the FS electrodes. Simulation and experimental results indicate that the FS electrode traces can be stretched over 20% (strain levels of skin: 10–20%), where the maximum principal elastic strain in the metals is 11.24% (fracture strain of Cu: 33.3%). The corresponding optical microscope (OM) images of the FS mesh electrodes show that the FS electrodes do not show any cracks when the FS electrode trace is stretched by 20%. More details and figures can be seen in the supplementary information (Figs. S2–S5).

### Silent speech recognition

**Fig. 2:**
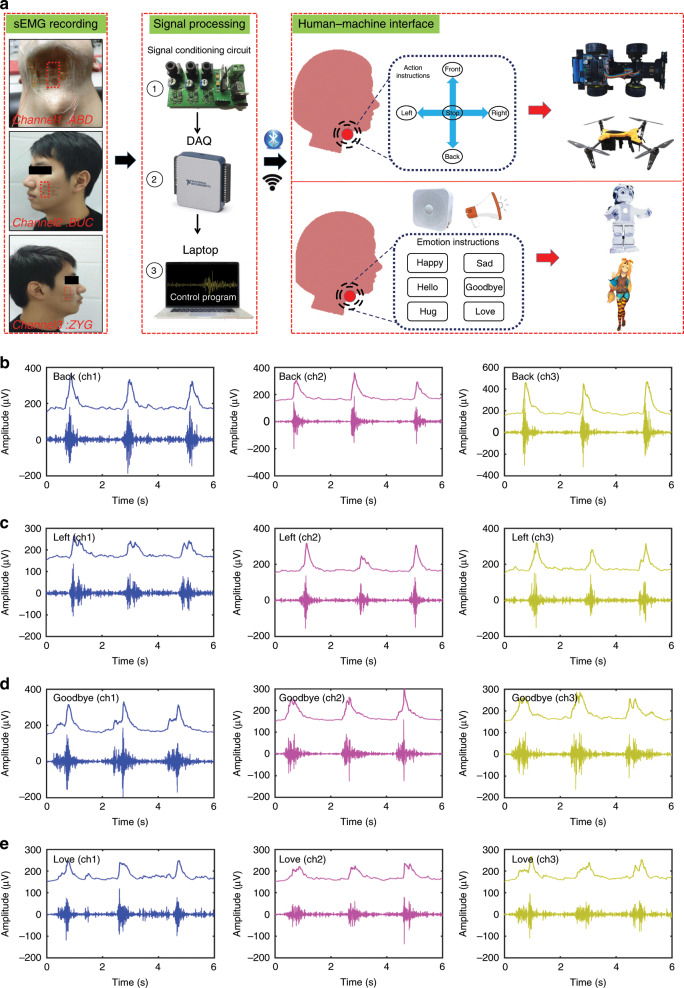
The intelligent silent speech recognition system for human–machine interface via sEMG. **a** Illustrations of the epidermal sEMG tattoo-like patch worn by a tester (three channels), and flow diagram of the sEMG system for intelligent silent speech recognition with a real-time voice synthesizer as the human–machine interface. **b–e** sEMG signals and their envelopes recorded from three muscle channels when the tester spoke the words “Back”, “Left”, “Goodbye”, and “Love”, respectively.

The flow diagram of the sEMG system for intelligent silent speech recognition and the human–machine interface are depicted in Fig. 2a. In the experiment, the epidermal sEMG tattoo-like patches were attached to a tester’s lower jaw and the left and right sides of the face, representing three different sEMG channels from three targeted muscle groups. The proposed silent speech recognition system measures the sEMG signal in a differential electrode configuration. Two measuring electrodes (MEA) of the patch were placed along the target muscle. Thereafter, the differential activation between the two measuring electrodes against the reference electrode (REF) was transmitted into the sEMG conditioning circuit (ZTEMG-1300, Qingdao, China) to reduce the interference caused by noise sources, such as power supply interference and movement artifacts, which can increase the signal-to-noise ratio (SNR) validly. The sEMG signals were recorded at 2000 Hz with a 16-bit resolution by using a data acquisition unit (NI USB-6003, Austin, Texas) and filtered with a band-pass filter with cutoff frequencies of 10–1000 Hz when the tester spoke silently. Then, the sEMG signals were converted into digital signals and analyzed by using MATLAB (The Mathworks, Inc., Natick, MA).

For patients with loss of voice, their action and emotion intentions are very important and must be expressed properly in their daily lives. Therefore, we selected five frequently used words, i.e., Front, Back, Left, Right, and Stop, as the basic action instructions to express their daily action intentions. Meanwhile, some common words, i.e., Happy, Sad, Hello, Goodbye, Hug, and Love, were chosen as the emotion instructions that these patients could use to express their emotions or greetings. The anterior belly of digastric (ABD), buccinators (BUC), and zygmaticus (ZYG) were selected as the target muscle groups because of their close connections with the talking and swallowing actions (channel #1: ABD, channel #2: BUC, and channel #3: ZYG). Once the silent speech recognition system was set up, the tester was asked to carry out a series of tasks:

- Baseline: The tester was asked to remain still and breathe quietly for 5–10 s to keep the muscles relaxed.
- Action instructions (each action for 30 trials): The tester was asked to speak five action instructions (Front, Back, Left, Right, and Stop) silently.
- Emotion instructions (each action for 30 trials): The tester was asked to speak six emotion instructions (Happy, Sad, Hello, Goodbye, Hug, and Love) silently.

Figure 2b, c shows the spectra and envelopes of the recorded sEMG signals from the three muscle channels when the tester spoke the action instructions (Back and Left). Similarly, the recorded spectra and envelopes of the sEMG signals for the emotion instructions (Goodbye and Love) are depicted in Fig. 2d, e. The other sEMG signals for the action instructions (Front, Right, and Stop) and emotion instructions (Sad, Hello, Happy, and Hug) are shown in the supplementary information, Figs. S4 and S5.

**Fig. 3:**
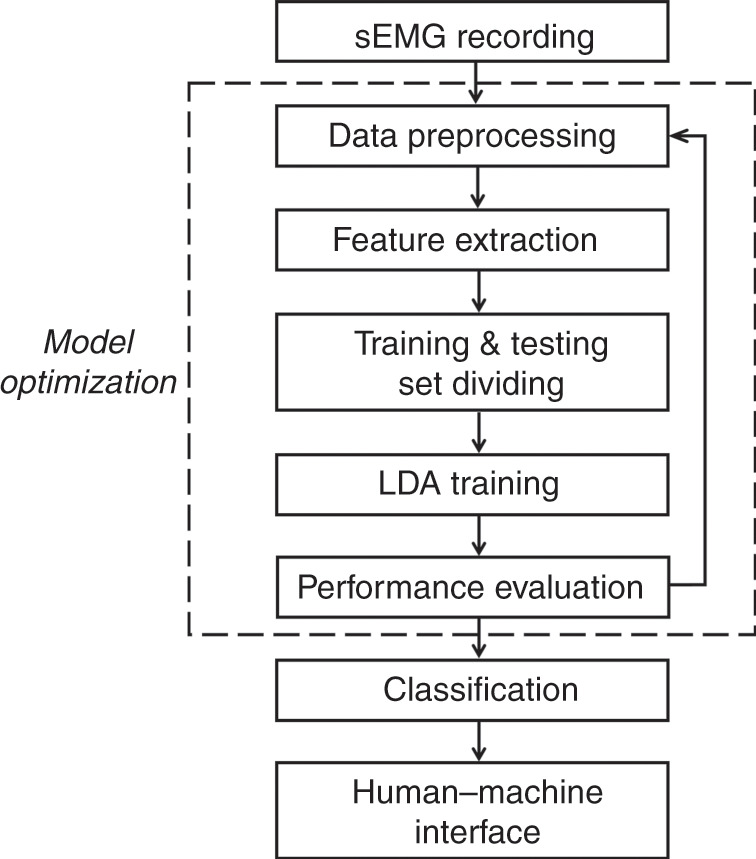
Procedure of the proposed silent speech identification method.

The procedure of the proposed silent speech recognition method based on the wavelet decomposition and pattern recognization is illustrated in Fig. 3. First, the sEMG signals from three muscle channels are recorded by the epidermal tattoo-like patch. Then, features are extracted from the sEMG signals, which have been preprocessed to construct a data set. The data set is then divided into training and testing sets, in which the training set is used to train the linear discriminant analysis (LDA) model in the training stage and the testing set is input to the trained model to evaluate the identification performance. According to the evaluation results, feature and preprocessing methods are applied to optimize the LDA model again. Finally, the original sEMG signals are translated into control commands for HMI.

**Fig. 4:**
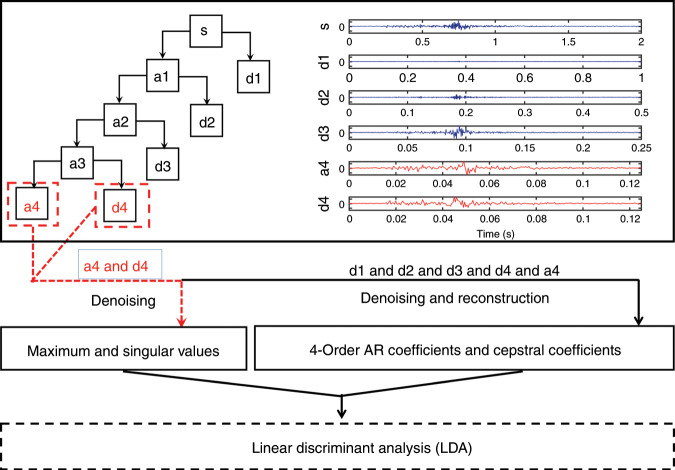
A four-layer hierarchy of the wavelet decomposition and basic framework of sEMG processing.

The implementation of the wavelet decomposition and pattern recognition for data processing can be further elaborated, as shown in Fig. 4. In the surroundings of the target muscle, the common mode noise and 50-Hz power line interference will reduce the quality of the sEMG signal from the targeted muscles. The other high-frequency noise within the biophysical bandwidth comes from movement artifacts that change the skin–electrode interface, muscle contraction or electromyographic spikes, respiration (which may be rhythmic or sporadic), electromagnetic interference, and so on. Therefore, noise reduction is necessary before feature extraction. In this work, the wavelet transform method is used for noise reduction because of its great time–frequency localization characteristics and unique advantages in addressing nonstationary time-varying signals. In addition, the wavelet transform can better preserve the abrupt part of the signal and useful information while filtering out the signal noise.

The original sEMG signal {s} is decomposed by the db4 wavelet; after four levels of decomposition, five-set coefficients are produced: {d1, d2, d3, d4, a4}. a4 is the low-frequency coefficient segment. d1, d2, d3, and d4 are the high-frequency coefficient segments. The waveforms of the wavelet coefficients a4 and d4 are similar to those of the original signal {s}, which means the wavelet coefficients {d4, a4} contain the most valid information of the original signal.

**Fig. 5:**
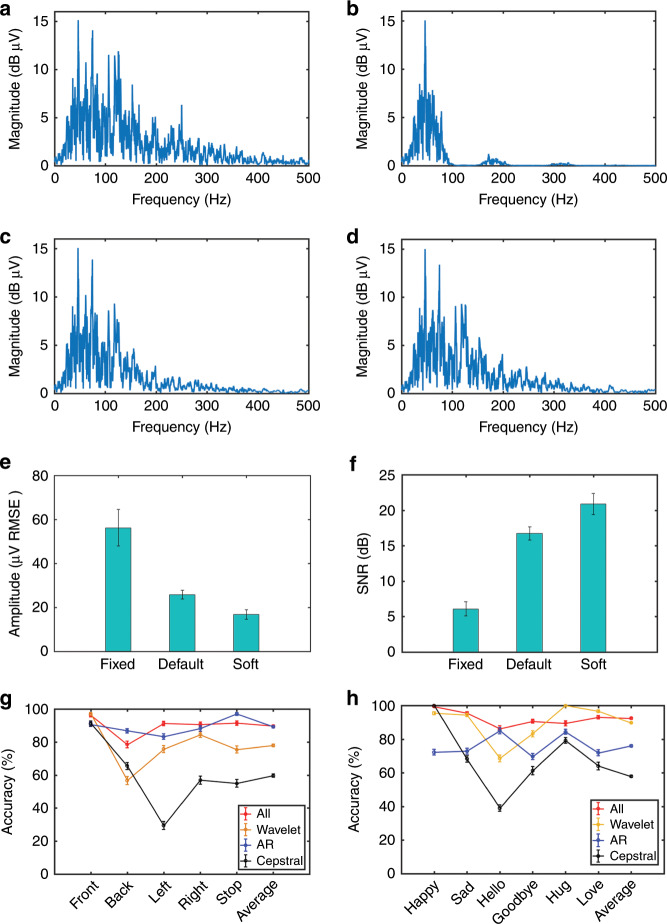
Evaluation results obtained through three different approaches of denoising and wavelet decomposition. **a** Spectrogram of the original signal. **b–d** Spectrograms of the signal after fixed threshold denoising, default threshold denoising, and soft threshold denoising, respectively. **e**, **f** Root mean squared error (RMSE) and signal-to-noise ratio (SNR) of the sEMG signals using three denoising approaches. **g**, **h** Comparison of recognition accuracies of pattern recognition for action and emotion instructions, respectively, through different features.

The obtained wavelet coefficients are processed by three different threshold quantization functions, i.e., fixed threshold denoising, default threshold denoising, and soft threshold denoising, and then the signal is reconstructed. As shown in Fig. 5a–d, the default threshold denoising and soft threshold denoising can preserve the active ingredients of the sEMG from 50 to 200 Hz, which is the effective frequency bandwidth of sEMG. With the introduction of two quantitative indicators, the SNR and root mean square error (RMSE), as the criteria to pass judgment on the denoising effect, soft threshold denoising shows a better SNR (20.87 ± 1.47 dB) and lower RMSE (16.82 ± 2.1 μV), as shown in Fig. 5e, f. In the feature extraction process, a feature vector with a length of ten is constructed by extracting the maximum and singular values from each wavelet coefficient {a4, d4}, extracting the four-order autoregressive (AR) model coefficients, and selecting the first two cepstral coefficients to characterize each trial.

In this study, 150 feature vector sets were recorded from the five action instructions, and 180 feature vector sets were recorded from the six emotion instructions (each instruction for 30 trials). Tenfold cross-validation was applied. Ninety percent of the feature vector sets were used for the training of the LDA, with the others used for testing. Figure 5g, h shows the accuracy comparison of pattern recognition by using different features. The average accuracy of pattern recognition by using cepstral coefficients as feature vectors is lower than that using other features in both the action and emotion instructions, which indicates that cepstral coefficients do not apply to this experiment. Hence, in this study, the AR model coefficients and the maximum and singular values of the wavelet coefficients were combined to construct a feature vector, aiming to reduce its dimensions.

**Fig. 6:**
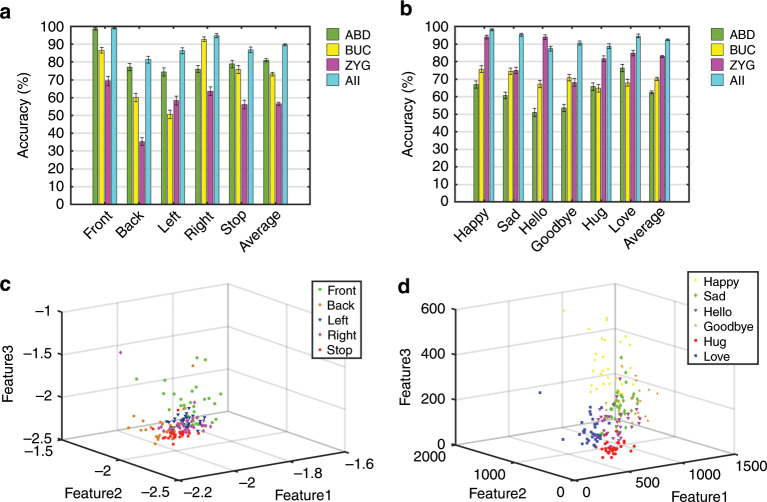
Recognition rates of silent speech recognition. **a, b** Recognition rates of three single channels and combined channels for five action and six emotion instructions, respectively. **c**, **d** Visualization of features from sEMG of five action and six emotion instructions, respectively.

The accuracies of the five action and six emotion instructions from the three individual channels and the combined channel are depicted in Fig. 6a, b. The results show that the average accuracy of the action instructions (Front, Back, Left, Right, and Stop) of the combined channel can reach up to 89.6 ± 0.6% and that the best accuracy of a single channel is 80.9 ± 0.9%. The average accuracy of the emotion instructions (Happy, Sad, Hello, Goodbye, Hug, and Love) of the combined channel can reach up to 92.7 ± 0.5%, and the best accuracy of a single channel is 81.9 ± 0.6%. Figure 6c, d shows the visualization of features from the sEMG of the six emotion and five action instructions. The detailed accuracies of these three muscle channels are presented in Tables S2 and S3 (Supplementary Information). It can be seen from the figure that the ABD muscle channel achieves higher accuracy in terms of the action instructions than do the other two channels, while the ZYG muscle channel shows the best accuracy in terms of the emotion instructions among the three channels. This result may be due to the individual differences in terms of the muscle group and the pronunciation habit of the tester during silent speech. It is obvious that an increase in the number of channels can increase the accuracy. However, considering that an increase in the number of channels is at the expense of the comfort of the testers, we select the sEMG of the ABD channel (for action instructions) and the ZYG channel (for emotion instructions) as the control signal sources for the later HMI process.

### Human–machine interface

**Fig. 7:**
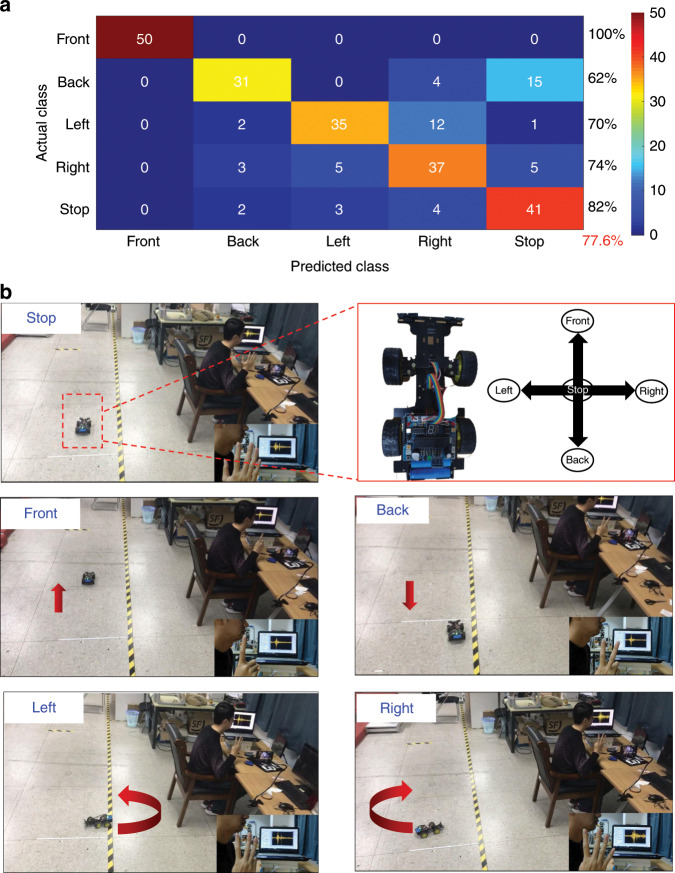
Flowchart of the human–machine interface (HMI) for controlling an intelligent car via sEMG signals recorded by the epidermal sEMG tattoo-like patch. **a** Confusion matrix of the ABD channel for the action instructions. **b** Intelligent car controlled by the sEMG signals of the action instructions from the ABD channel.

These sEMG signals are binned into discrete commands using the LDA classification algorithm. The accuracy of this classifier can then be visualized utilizing a confusion matrix (Fig. 7a), in which the columns and rows represent the predicted class and the actual instructions (“actual class”), respectively. As in the figure, the five action instructions correspond to distinct commands: Front, Back, Left, Right, and Stop. As an example, the success rate for the Front command is 100% for 50 trials. The overall accuracy of all five classifications is 77.6%. The five action instructions control the intelligent car with regard to five different motions, i.e., Front: “move forward”, Back: “move backward”, Left: “turn left”, Right: “turn right”, and Stop: “stop moving”. As demonstrated in Fig. 7b, the movement of the intelligent car can be successfully controlled in this manner. The specific demo video can be found in the supplementary information (Movie S1).

**Fig. 8:**
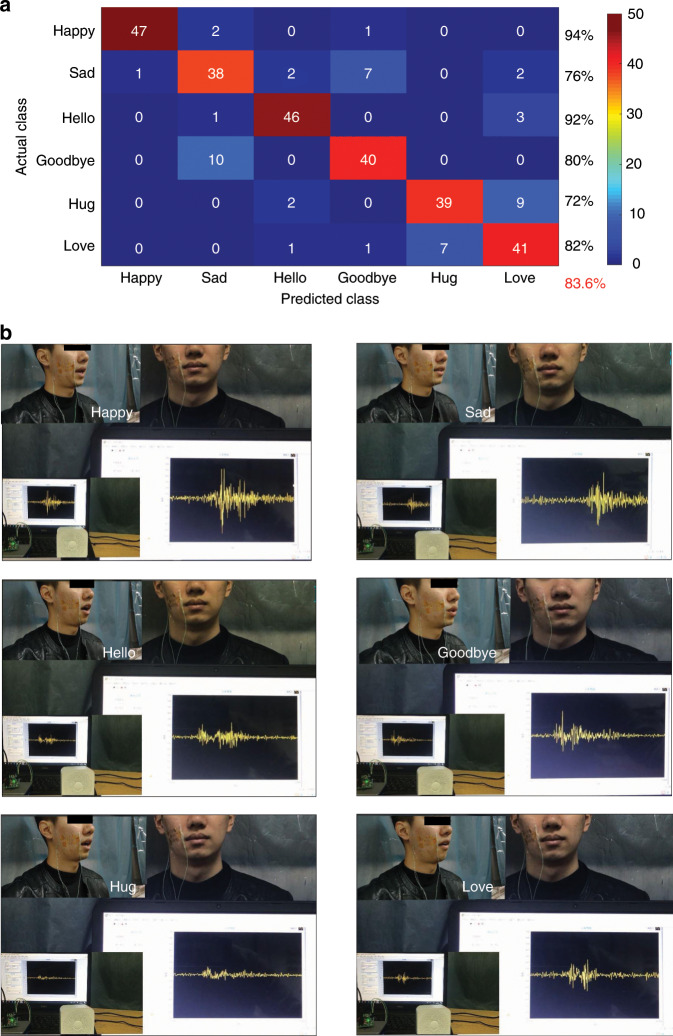
Flowchart of the human–machine interface (HMI) for the voice synthesis of the Bluetooth speaker via sEMG signals recorded using the epidermal sEMG tattoo-like patch. **a** Confusion matrix of the ZYG channel for the emotion instructions. **b** Voice synthesis of a Bluetooth speaker by sEMG signals of the emotion instructions from the ZYG channel (the vignettes at the top left indicate the mouth types when the tester spoke the six different emotion instructions).

Similarly, the accuracy of the emotion instructions can be visualized by a confusion matrix (Fig. 8a). As shown in the figure, the six emotion instructions correspond to distinct commands: Happy, Sad, Hello, Goodbye, Hug, and Love. The success rate for the command “Hello” is 94% for 47 trials. The overall accuracy of all six classifications is 83.6%. The six emotion instructions were synthesized and broadcasted simultaneously by a Bluetooth speaker. As depicted in Fig. 8b, the voice synthesis of the Bluetooth speaker can be successfully controlled in this manner (the vignettes at the top left indicate the mouth types when the tester spoke the six different emotion instructions). The specific demo video can be found in the supplementary information (Movie S2).

**Fig. 9:**
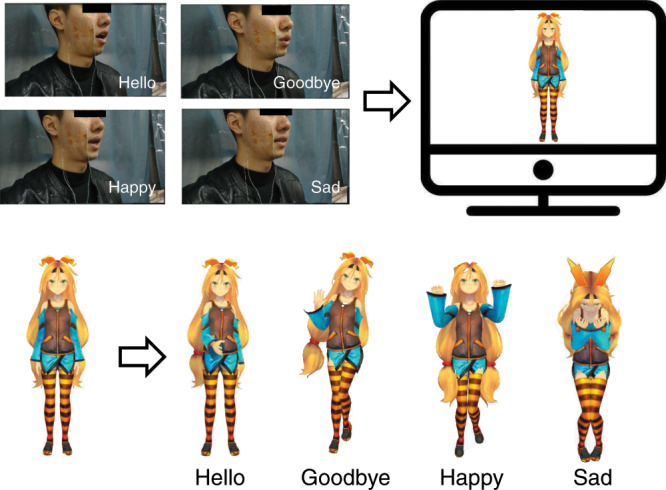
Flowchart of the augmented reality (AR) interaction via sEMG signals recorded using the epidermal sEMG tattoo-like patch.

In addition, it may be slightly insufficient to express emotions simply by relying on simple voice synthesis for people who have lost their voice. Therefore, we created a virtual character to represent the patient that could pronounce the patient’s words and make some body movements to better express their motions. As shown in Fig. 9, when the tester spoke “hello” silently, the virtual animated character would raise his hand to express the intention to shake hands. Then, the virtual animated character would make a goodbye gesture when the tester spoke “goodbye”. Finally, the virtual animated character would bounce to express joy and cry while hiding his face to express sadness when the tester spoke “happy” and “sad”, respectively. The specific demo video can be found in the supplementary information (Movie S3).

## Conclusions

In summary, an imperceptible epidermal sEMG tattoo-like patch is developed to monitor sEMG signals generated by the muscles of the face and the jaw. The epidermal sEMG electrodes manufactured by the MEMS process combined with a sterile wound dressing provide excellent flexibility and viscosity to adapt to the strain of skin. This tattoo-like patch shows excellent performance in recording the sEMG of three muscle channels when a tester speaks silently. The collected signals are processed by wavelet decomposition and a machine-learning algorithm to identify the action and emotion instructions of the tester. The results indicate that the average accuracy of the action instructions (Front, Back, Left, Right, and Stop) from the combined channel can reach up to 89.6% and that the best accuracy of a single channel is 80.9%. For the emotion instructions (Happy, Sad, Hello, Goodbye, Hug, and Love), the average accuracy of the combined channel can reach up to 92.7%, and the best accuracy of a single channel is 81.9%. In the future, such flexible and functional tattoo-like patches will have great potential in intelligent speech recognition and voice synthesis, which is of great importance to help patients who have lost their voice to express their intentions by an HMI, such as wheelchair control, a human–robot interface, virtual reality interaction, etc. The device also offers expansion of the potential application to artificial throat and healthcare monitoring. In the future, thinner and more integrated electronics patches will show great potential in medical treatment, biomonitoring, sensing, etc.

## Experimental section

### Fabrication of the epidermal sEMG electrodes

The fabrication process began with the preparation of a glass substrate to facilitate the delamination of electrode patterns. It was followed by spin-coating polydimethylsiloxane (PDMS, 100 μm in thickness) onto the temporary substrate (mixed at a 10:1 ratio, 3000 rpm for 30 s, at 110 °C for 2 h) and exposed to oxygen plasma to enhance the vitality of the surface. A thick layer of PI (2.4 μm in thickness) was made for the protective layer (2000 rpm for 60 s, at 150 °C for 4 min and at 210 °C for 1 h). Then, a thick layer of Cu (400 nm in thickness) was deposited by electron beam evaporation onto the PI. The electrode and interconnect structure were defined by photolithography and etching (CH_3_COOH:H_2_O_2_:H_2_O = 1:2:10). Then, a second PI layer (2.4 μm in thickness) and silicon oxide layer (SiO_2_, 200 nm in thickness) were used to cover the entire structure. Next, photolithography, reactive ion etching (RIE), and oxygen plasma etching were conducted to pattern the layers of PI in a geometry matched to the metal traces and exposed the sEMG electrodes and connection pads (20 Sccm O_2_, 80 mT, 200 W for 60 min). The residue SiO_2_ mask was removed by using buffered oxide etchant (BOE, 1:20). Finally, the wafer was soaked in dilute hydrochloric acid (HCl, 3% concentration) for 30 s and cleaned by flowing water to remove the copper oxide layer.

### Wavelet decomposition

Continuous sampling of the continuous signal *f(t)* can yield the corresponding discrete signal *f(n)* (*n* = 0, 1, ……*N*–1). The wavelet transform is defined as

$$W_f(j,\,k) = 2^{ - \frac{j}{2}}\mathop {\sum}\limits_{n = 0}^{N - 1} {f(n)\psi (2^{ - j}n - k)}$$

where $\psi ({\it{2}}^{ - j}n - k)$ is the wavelet function and *W*_*f*_(*j*, *k*) is the wavelet coefficient. The wavelet transform is implemented by the Mallat algorithm as follows:

$$\begin{array}{l}S_f(j + i,\,k) = S_f(j,k) ^\ast h(j,\,k)\\ W_f(j + i,\,k) = W_f(j,\,k) ^\ast g(j,\,k)\end{array}$$

Accordingly, the refactoring formula is

$$S_f(j - i,\,k) = S_f(j,\,k) ^\ast \hat h(j,\,k) + {W_{f(j,\,k)}} ^\ast \hat g(j,\,k)$$

where *h*(*j*, *k*) denotes the low-pass and high-pass filters corresponding to the scaling function, *g*(*j*,*k*) denotes the low-pass and high-pass filters corresponding to the wavelet function Ψ (*x*), and “^*^” implies conjugation.

### Experiments on human subjects

All experiments were approved by the human protection program at Soochow University.

## Supplementary information

Supplementary information

Augmented reality interaction

Bluetooth speaker controlling (Online)

Intelligent car controlling (Online)
