# Supplementary material for: An epidermal sEMG tattoo-like patch as a new human–machine interface for patients with loss of voice
Source: Microsyst Nanoeng. 2020 Mar 9;6:16. doi: 10.1038/s41378-019-0127-5 (PMC8433406; doi:10.1038/s41378-019-0127-5)
Supplement: Supplementary file 1 — Supplementary information [file 41378_2019_127_MOESM1_ESM.docx]

**Supplementary Information**

**An epidermal sEMG tattoo-like patch as a new human–machine interface for patients with loss of voice**

*Huicong Liu^1^, Wei Dong^1^, Yunfei Li^1^, Fanqi Li^1^, Jiangjun Geng, Minglu Zhu^2,3^,*

*Tao Chen^1^, Hongmiao Zhang*^1^, Lining Sun*^1^ and Chengkuo Lee*^2,3^*

**S1. Fabrication of the epidermal sEMG electrode**


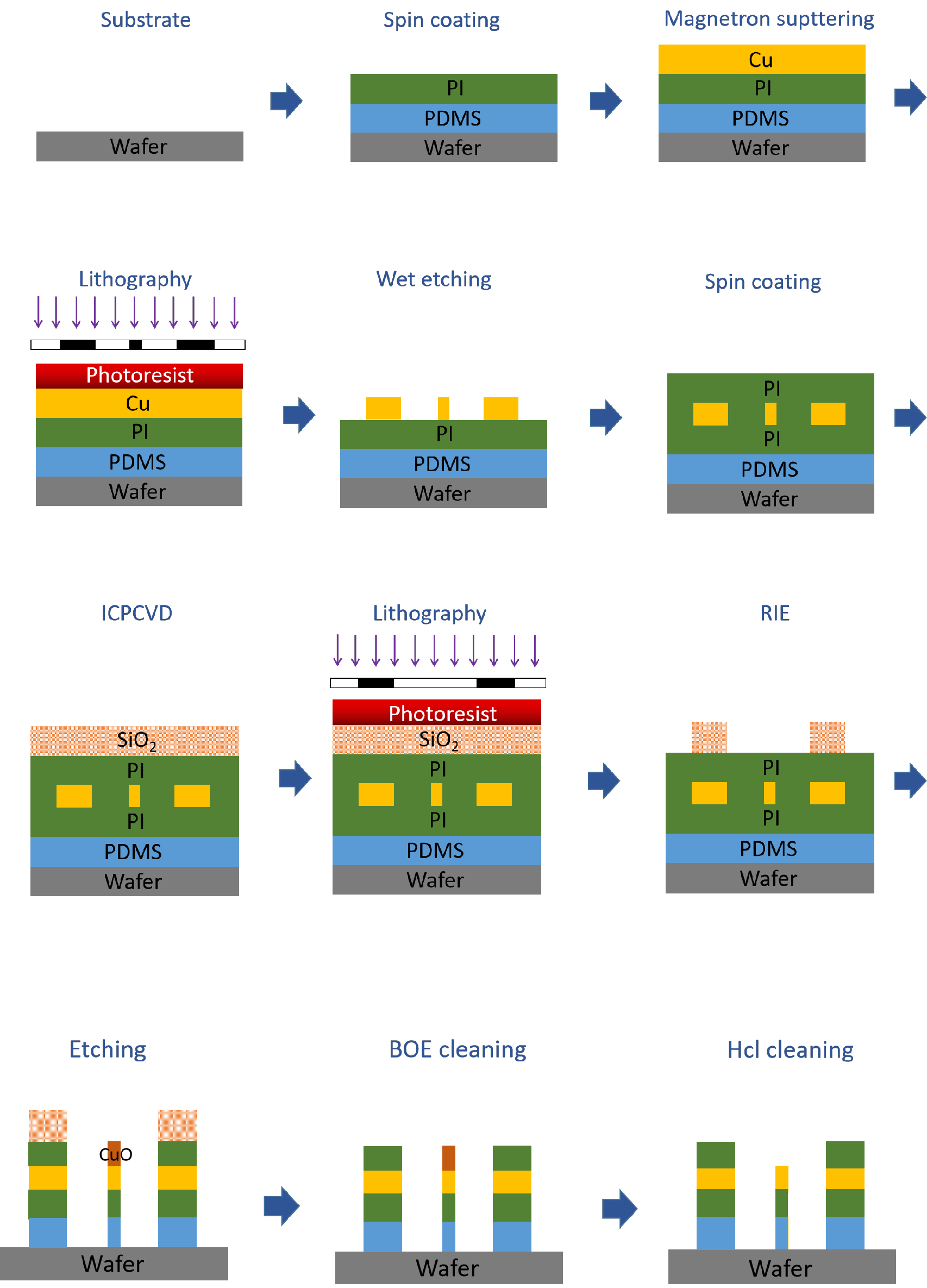


**Fig. S1** Device structure and fabrication steps of the epidermal sEMG electrode.

**S2. Image of the sterile wound dressing**


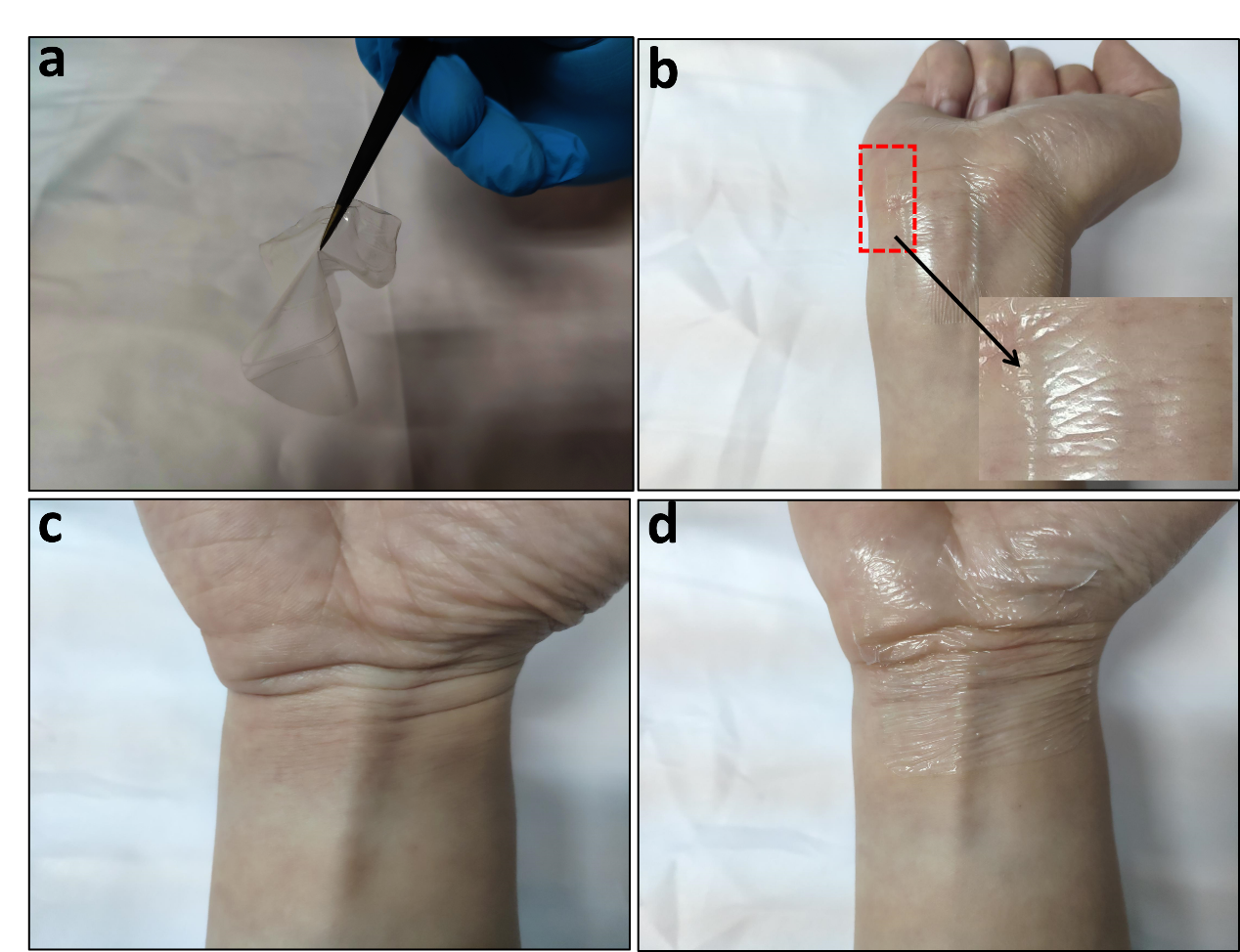


**Fig. S2** Image of the sterile wound dressing. a) Image of the wrist with a sterile wound dressing, along with zoomed view indicating the film conforming to skin groves. b) Images of the curved wrist and the sterile wound dressing attached to the curved wrist.

**S3. Photographs showing the excellent flexibility and adhesiveness of the epidermal sEMG electrode patch.**


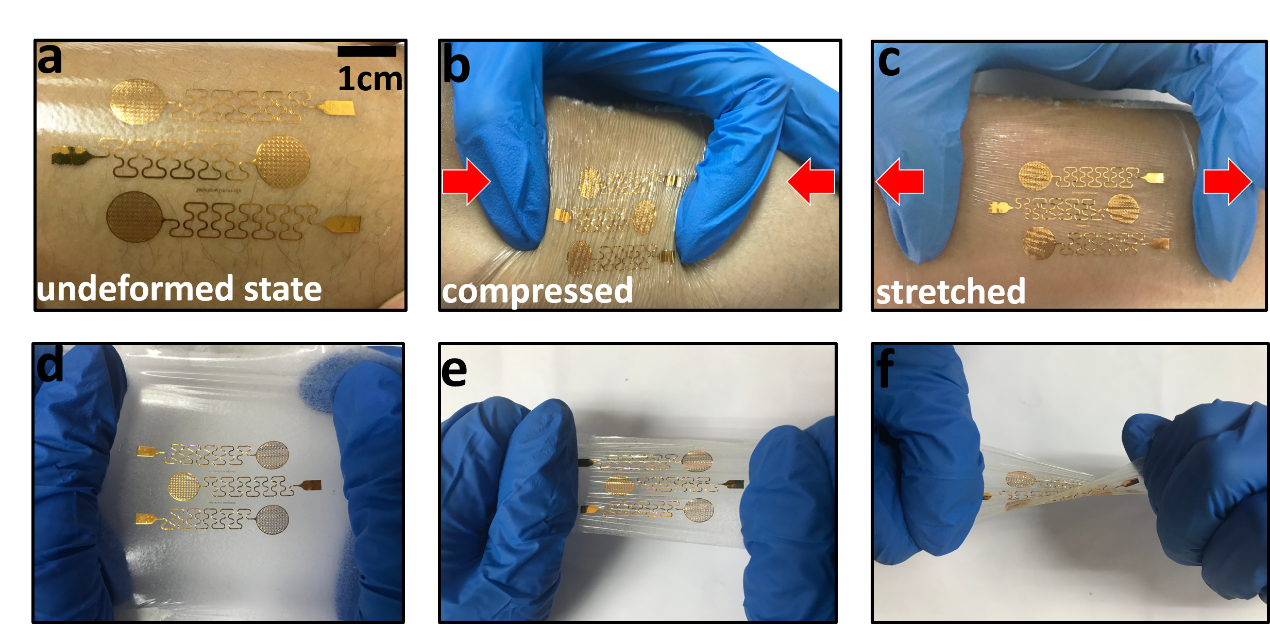


**Fig. S3** Photographs showing the excellent flexibility and adhesiveness of the epidermal sEMG electrode patch. a-c) The epidermal sEMG electrode patch mounted on the forearm and encapsulated with a layer of sterile wound dressing under compression and extension of the skin. d) Picking up the epidermal sEMG electrode patch normally. e) Stretching the epidermal sEMG electrode patch. f) Reversing the epidermal sEMG electrode patch.

The bending energy of a traditional EES depends on the peeling strength of the EES electrode (modulus of Au: 97GPa, thickness including encapsulating PI layers: 0.8μm) and the membrane (modulus of the membrane: 69 kPa; Ecoflex). The elastic energy of the skin is mainly determined by the wavelength (140 μm) and modulus (≈130 kPa) of the skin. Assuming that the contact adhesion is dominated by the interface between the EES, membrane, and the skin, the effective work of adhesion is ≈0.25 Nm^-1^. Conformal contact can be achieved when the adhesion energy is larger than the sum of the bending and the elastic energy.^[1]^ Compared with EES, the epidermal sEMG tattoo-like patch is of higher modulus (modulus of Cu: 119 GPa, thickness including PI layers: 2.8 μm), but the peeling strength of the selected sterile wound dressing is more than 1000 Nm^-1^, which far meets the demand. Adhering the epidermal sEMG tattoo-like patch to the forearm is depicted in **Figure** S3a, because of great peeling strength, the epidermal sEMG tattoo-like patch still maintains great conformal contact during stretching and compression as illustrated in **Figure** S3b and 3c. Apart from peeling strength, experiments on EES in recent years have shown that EES built using membranes with thicknesses smaller than the critical membrane thickness (≈25μm) will establish conformal contact to the skin.^[2]^ The ultra-thin thickness of the selected sterile wound dressing is less than 25μm. The ultra-thin thinness of the sterile wound dressing (about 5.84μm) also brings excellent flexibility and toughness to the entire device (see in the **Figure** S2). **Figure** S3d, 3e and 3f show the tattoo-like patch is able to withstand any normal picking, stretching and twisting.

The sterile wound dressing makes excellent conformal contact with wrist, covering wrinkles and pits of the skin surface well. In other words, the sterile wound dressing can be adhered to skin firmly and reduce discomfort when wearing because of its ultra-thinness and excellent peeling strength. This performance is vital when the epidermal sEMG electrode patch is attached to the face and jaw for the demonstration of silent speech recognition.

**S4. Simulation and experimental testing of tensile properties of the epidermal sEMG electrode patch.**


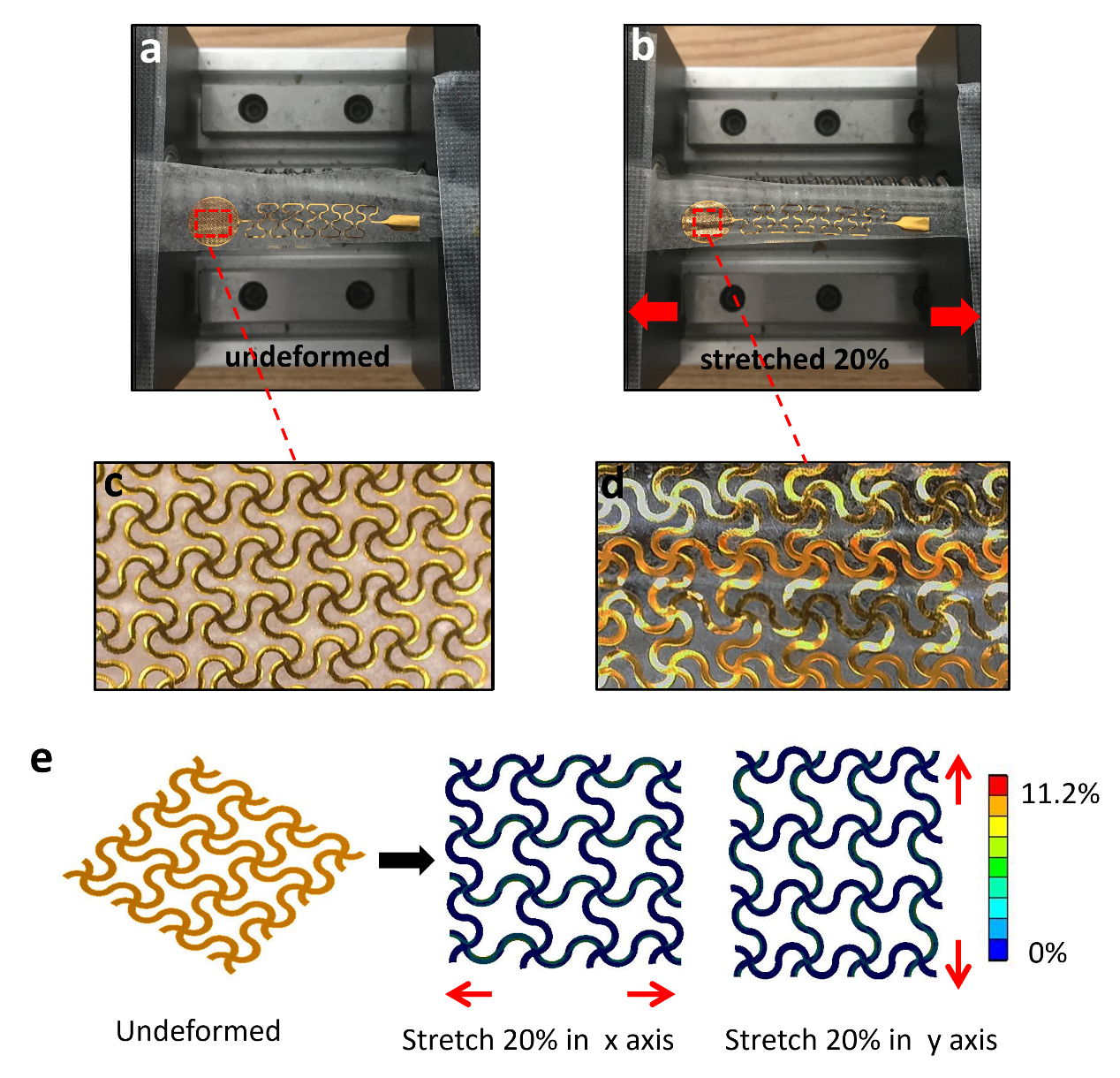


**Fig. S4** Simulation and experimental testing of tensile properties of the epidermal sEMG electrode patch. a, b) Pictures of an epidermal sEMG electrode patch undeformed and stretched over 20% by using a mechanical stage. c, d) OM images of the FS mesh electrode undeformed and stretched over 20%. e) Finite element analysis of an EES comprised of a FS mesh with 100 μm widths, under mechanical stretching over 20% along the x and y directions.

**S5. The simulation of mechanical tensile properties of the double-wave wires.**


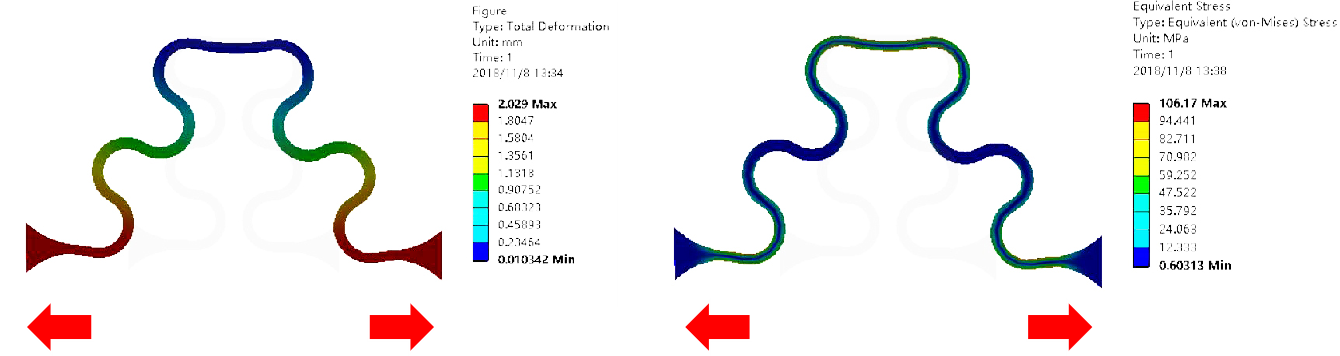


**Fig. S5** The simulation of mechanical tensile properties of the double-wave wires. When the deformation of the double-wave wire reaches 100%, the maximum tensile stress (106.17 MPa) is less than the tensile strength of PI (130 MPa).

| Material | Density | Young's modulus | Poisson's ratio | Thickness | Displacement |
| --- | --- | --- | --- | --- | --- |
| PI | 1300kg/m^3^ | 3.1*10^9^ Pa | 0.37 | 4.8μm | 2mm |

**Table S1.** Material parameters in the simulation

The bare FS mesh copper electrodes enable direct electrical coupling to the skin, which requires the FS mesh electrode not only providing great conformal contact, but also allowing sufficient deformation to adapt to the movement of the skin. To reduce the failure rate during fabrication and wearing test, the FS mesh electrode is designed with the wire width of 0.1mm and the radius of curvature of 0.2mm. The FS mesh electrodes minimizes the contact impedance by providing large areal coverage. **Figure** S4a and S4b are the epidermal sEMG tattoo-like patch undeformed and stretched over 20% by using a mechanical stage and **Figure** S4c and S4d are the corresponding optical microscope (OM) images of the FS mesh electrodes. **Figure** S4e is the image of using the finite element method (FEM) to simulate the deformation and stress distribution of the FS electrodes. Simulation and experimental results indicate that the FS electrode traces can be stretched over 20% where the maximum principal elastic strain in the metals is 11.24% (fracture strain of Cu: 33.3%). When the FS electrode trace is stretched by 20%, the electrode does not show any cracks. Such tensile property exceeds strain levels of skin (10%-20%) and satisfies the application requirement. It is worth mentioned that the double-wave wires can be stretched more than 100% by simulation, which can provides better tensile property for the patch. (**Figure** S5 and **Table** S1). Experimental determination of contact impedance involves measurements, using the compact Inductance Resistance Capacitance (LCR) Meter (TH283X V1.6, Tonghui Company, Chang zhou), which indicates that the epidermal sEMG tattoo-like patch shows remarkably lower contact impedance(≈36.4 KΩ) than that of the gel electrodes(≈40.6 KΩ).

**S6. Applications of the epidermal sEMG electrode patch for sEMG signal sensing on three muscle channels when tester spoke action instructions silently**


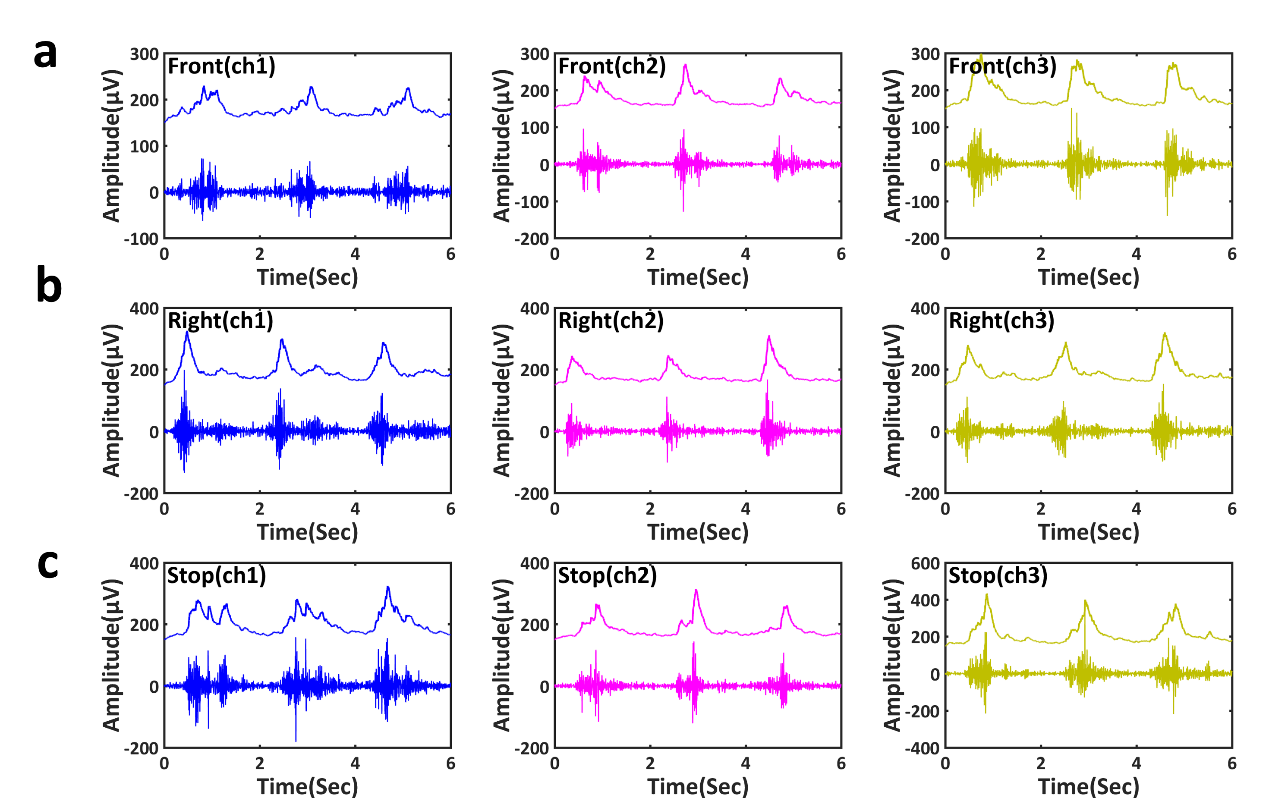


**Fig. S6** Applications of the epidermal sEMG electrode patch for sEMG signal sensing on three muscle channels when tester spoke action instructions silently. a-c) the sEMG singles and their envelopes recorded from three muscle channels when the tester spoke Front. Right and Stop, respectively.

**S7. Applications of the epidermal sEMG electrode patch for sEMG signal sensing on three muscle channels when tester spoke emotion instructions silently.**

**
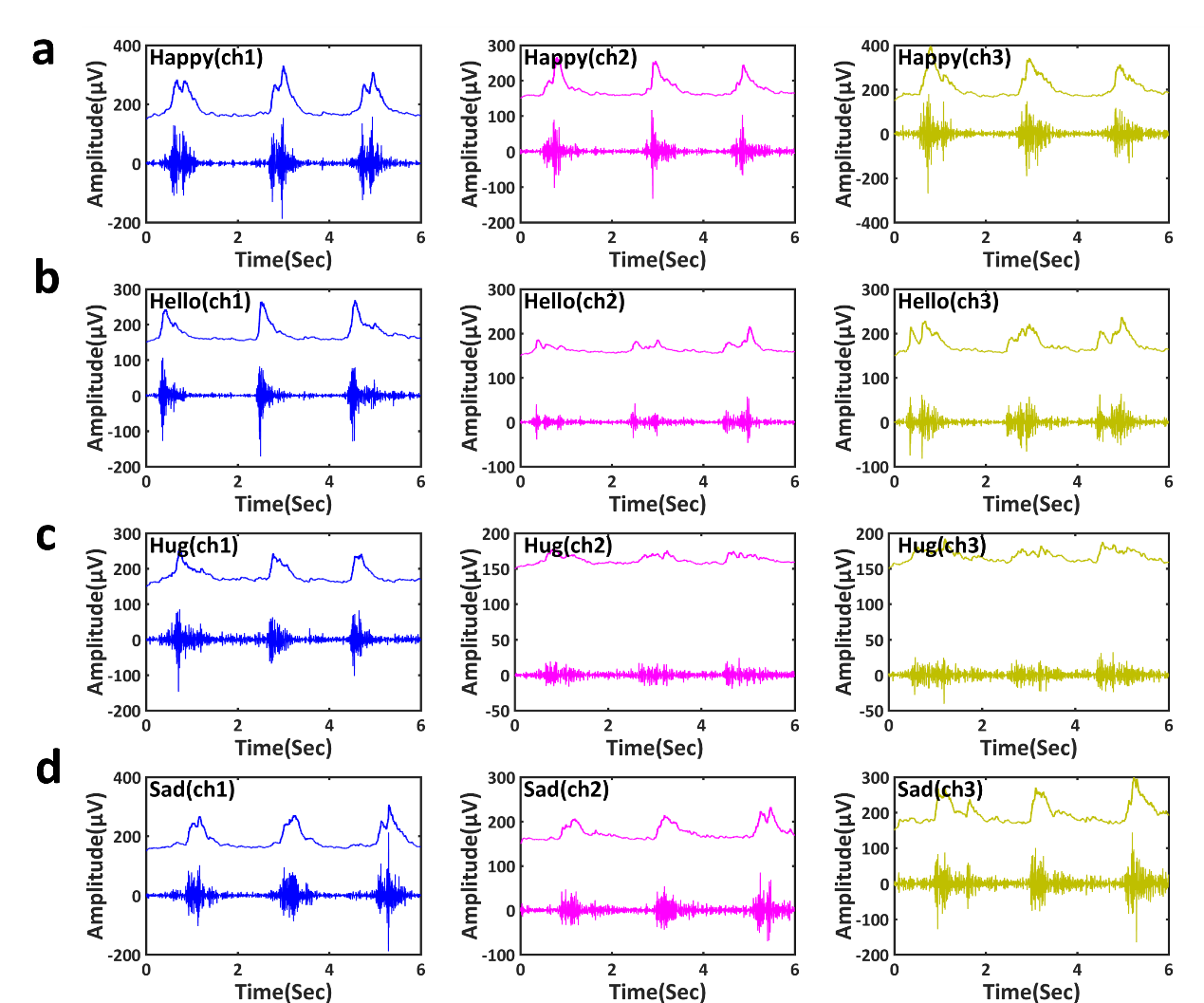
**

**Fig. S7** Applications of the epidermal sEMG electrode patch for sEMG signal sensing on three muscle channels when tester spoke emotion instructions silently. a-d) the sEMG singles and their envelopes recorded from three muscle channels when the tester spoke Happy. Hello. Hug and Sad, respectively.

|  | Front （%） | Back （%） | Left （%） | Right （%） | Stop （%） | Average（%） |
| --- | --- | --- | --- | --- | --- | --- |
| ABD | 98.5±0.7 | 77.0±2.0 | 74.3±2.2 | 76.0±1.9 | 78.8±2.1 | 80.9±0.9 |
| BUC | 86.5±1.6 | 60.0±2.4 | 50.5±2.3 | 92.8±2.2 | 75.8±2.2 | 73.1±0.9 |
| ZYG | 69.3±2.5 | 35.3±2.2 | 58.3±2.5 | 63.5±2.4 | 56.0±2.4 | 56.5±1.0 |
| ALL | 99.0±0.4 | 81.3±1.9 | 86.3±1.7 | 94.8±1.6 | 86.8±1.6 | 89.6±0.6 |

**Table S2.** Each detailed accuracy of three muscle channels about five action instructions.

|  | Happy（%） | Sad （%） | Hello（%） | Goodbye（%） | Hug （%） | Love（%） | Average（%） |
| --- | --- | --- | --- | --- | --- | --- | --- |
| ABD | 66.4±1.9 | 61.0±2.0 | 51.2±2.4 | 50.8±2.2 | 64.6±2.0 | 72.0±2.1 | 61.0±0.8 |
| BUC | 76.8±1.8 | 69.2±1.9 | 66.2±1.8 | 68.6±2.2 | 66.8±1.9 | 69.8±2.3 | 69.5±0.7 |
| ZYG | 91.0±1.4 | 74.8±1.7 | 92.4±1.2 | 72.4±2.1 | 77.4±1.7 | 83.8±1.6 | 81.9±0.6 |
| ALL | 98.6±0.3 | 95.8±0.9 | 86.2±0.9 | 92.0±1.2 | 90.2±1.4 | 93.4±1.1 | 92.7±0.5 |

**Table S3.** Each detailed accuracy of three muscle channels about six emotion instructions.

**References**

[1] Jeong, J. W. et al. Materials and optimized designs for human‐machine interfaces via epidermal electronics. Adv. Mater. **25**, 6839-6846 (2013).

[2] Yeo, W. H. et al. Multifunctional epidermal electronics printed directly onto the skin. Adv. Mater. **25**, 2773-2778 (2013).

# **Supplementary Movies**

# **Movie S1.** Intelligent car controlling (Online).

**Movie S2.** Blue tooth speaker controlling (Online).

**Movie S3.** Augmented reality interaction (Online).
